# A cost-effective and environmentally sustainable process for phycoremediation of oil field formation water for its safe disposal and reuse

**DOI:** 10.1038/s41598-019-51806-5

**Published:** 2019-10-23

**Authors:** Bhaskar Das, Suresh Deka

**Affiliations:** Environmental Biotechnology Laboratory, Life Sciences Division, Institute of Advanced Study in Science and Technology, Guwahati, Assam India

**Keywords:** Microbiology, Environmental sciences

## Abstract

High volumes of formation water comprising of complex mixture of hydrocarbons is generated during crude oil exploration. Owing to ecotoxicological concerns, the discharge of the formation water without remediation of hydrocarbonaceous pollutants is not permitted. Keeping this into mind, we carried out phycoremediation of hydrocarbons in formation water so that it can be safely discharged or re-used. For this, a native algal species was isolated from formation water followed by its morphological and 18S ribosomal RNA based identification confirming the algal isolate to be *Chlorella vulgaris* BS1 (NCBI GenBank Accession No. MH732950). The algal isolate exhibited high biomass productivity of 1.76 gm L^−1^ d^−1^ (specific growth rate: 0.21 d^−1^, initial inoculum: 1500 mg L^−1^) along with remediation of 98.63% petroleum hydrocarbons present in formation water within 14 days of incubation indicating an efficient hydrocarbon remediation process. Concomitantly, the hydrocarbon remediation process resulted in reduction of 75% Chemical Oxygen Demand (COD) load and complete removal of sulfate from formation water making it suitable for safe disposal or reuse as oil well injection water respectively. The present process overcomes the bottlenecks of external growth nutrient addition or dilution associated with conventional biological treatment resulting in a practically applicable and cost-effective technology for remediation of oil field formation water.

## Introduction

Formation water present in underground hydrocarbon bearing formations is brought to the surface along with crude oil during oil exploration and production. As per American Petroleum Institute, approximately 34.07 liters of formation water is produced per litre of oil produced from stripper oil well (oil well nearing the end of its economically useful life). The worldwide production of formation water is more than 77 billion barrel per year making the current oil industry looks more like a “water industry”^1–3^. This formation water is composed of a complex mixture of hydrocarbons. The discharge of the formation water without remediation of hydrocarbonaceous pollutants is a cause of grave concern since the hydrocarbons exhibit toxic effects as carcinogenicity, mutagenicity, teratogenicity and failure of vital body organs of aquatic organisms as well as humans^4,5^. Owing to ecotoxicological concerns associated with petroleum hydrocarbons, The Environment (Protection) Rules, 1986 of Ministry of Environment and Forests, India has set a permissible limit of 10 mg L^−1^ Total Petroleum Hydrocarbons (TPH) for disposal or less than 5 mg L^−1^ TPH for reuse (as oil well injection water) of formation water^6^. Oil field formation water contains TPH in the range of 28 to 126 parts per million (ppm) [ppm = mg L^−1^, mass per volume] which well beyond the permissible limits, set by the environmental regulatory agencies^7,8^. Keeping this in view, the petroleum industry requires to employ efficient and cost-effective technologies to reduce TPH in formation water below the permissible limit. Biodegradation encompassing its advantages of complete pollutant mineralization with no generation of secondary by-products that incurs additional treatment costs is currently being seen as an economic and eco-friendly technique for cleanup of hydrocarbon-polluted sites^9^. Hydrocarbon degrading bacterial strains are commonly applied for biological treatment of hydrocarbons. However, its application as an effective remediation strategy has been rendered questionable by its requirement of long adaptation periods as well as decreased oxygen saturation levels during hydrocarbon metabolism. Algal bioremediation on the virtue of its unique self-sustaining cycle could provide an efficient solution to overcome the above-mentioned drawbacks associated with conventional bioremediation strategies. Algae oxidize organic pollutants into non-harmful metabolites as CO_2_ and H_2_O by utilizing oxygen present in the surrounding environment. Algae perform photosynthesis for its growth, which requires CO_2_ and H_2_O. In turn, the oxygen released as a byproduct of photosynthesis could be utilized by algae for further pollutant oxidation, thus repeating the cycle^10^. These properties calls for significant attention to elucidate the potential of algae to serve as an effective phycoremediation strategy for removal of hydrocarbons in oil field formation water. Inspite of this, reports exploring the application of algae for treatment of hydrocarbon rich formation water is very severely limited. Talebi *et al*.^11^ attempted to treat diluted formation water by microalgal strain *Dunaliella salina*. They reported that *Dunaliella salina* could grow best when formation water is subjected to 1:1 dilution with sea water. *Dunaliella salina* could not degrade the hydrocarbons present in the diluted formation water, although removal of nitrogen (65%), phosphorus (40%) and heavy metals as Ni (90%) and Zn (80%) was achieved. It is to be noted that the algal strain *Dunaliella salina* was not isolated from a petroleum hydrocarbon contaminated site which may be a possible cause for its inability to remediate hydrocarbons in formation water. For an efficient bioremediation process, the microalgal strains should ideally be able to grow utilizing the hydrocarbonaceous pollutants as a carbon and energy source. This is quintessential to minimize the production of toxic degradation by-products as well as reduce the probability of isolates failing to survive at hydrocarbon contaminated samples due to lack of suitable growth substrates^12^. To address this, the present study attempts for the first time to isolate algal species native to oil field formation water in a bid to develop a microalgal process for remediation of petroleum hydrocarbon-rich formation water. Since the bioremediation process is based on the application of algal species native to oil field formation water, it is expected that the algal isolate will be able to meet its quintessential growth requirement of carbon and macro/micro-nutrients from the petroleum hydrocarbons and ionic composition found in formation water respectively. This implies that the present microalgal remediation process will overcome the conventional bottlenecks compromising the practical applicability of bioremediation strategies as requirement of external nutrient supplementation or dilution of the oil field formation water to maintain biomass growth.

## Results and Discussion

### Characteristics of the oil field formation water

The physico-chemical characterization of the formation water sample obtained from Oil and Natural Gas Corporation (ONGC) oil field is described in Table 1. The values obtained for various physico-chemical parameters of the formation water was compared with the environmentally permissible limits for safe disposal (on-shore or abandoned oil well disposal) or reuse as oil well injection water (Table 1). The comparison indicated that the concentration of TPH and COD in the formation water is well beyond the permissible limits prescribed by regulatory agencies for its safe disposal. Table 1 further brings to light that the TPH and sulfate load is far above the permissible limit for reuse as oil well injection water. The formation water to be reused as oil well injection water requires to be sulfate free or contain low sulfate concentration^13,14^. However, it is clearly evident from Table 1 that the values of other parameters as pH as well as heavy metals (Fe, Cu, Cr, Ni) except Zn is within the permissible disposal limits. The heavy metal composition of formation water is characterized by age of the wells as well as formation geology^15^. Apart from this, the ionic composition of formation water sample includes Na, Ca, Mg and N, all of which does not have any prescribed regulatory limit in formation water which could compromise its safe disposal or reuse. It is to be noted that the formation water exhibits the presence of significantly high concentration of Na (42.0972 mg L^−1^) and Ca (289.7853 mg L^−1^). This high concentrations of Na and Ca is a common characteristic of oil field formation water contributing mainly to its saline nature^15^.

**Table 1:** Characteristics of oil field formation water and the required specifications for its disposal and reuse.

Characteristics of oil field formation water	Values	Permissible Limits for formation water disposal and reuse		
		On-shore disposal (Environment (Protection Rules, 1986)	Disposal by reinjection into abandoned oil well (Environment Protection Rules, 1986)	Reuse by reinjection into oil reservoirs (Recommendation Standard and Analysis Method for the Clastic Rock Reservoir Injection Water Quality)
pH	8.35	5.5–9.0	No prescribed lower limit	No prescribed lower limit
TPH (mg L^−1^)	115	10	10	<5.0
SO_4_ (mg L^−1^)	48	1000	No prescribed lower limit	No prescribed lower limit. Low Sulfate/sulfate free water is desirable for reinjection (Jordan *et al*. 2008; Bader 2007)
COD (mg L^−1^)	316.80	100	No prescribed lower limit	No prescribed lower limit
Fe (mg L^−1^)	2.2746	3	No prescribed lower limit	No prescribed lower limit
Cu (mg L^−1^)	0.0066	0.05	No prescribed lower limit	No prescribed lower limit
Cr (mg L^−1^)	0.0393	0.1	No prescribed lower limit	No prescribed lower limit
Zn (mg L^−1^)	0.1763	0.1	No prescribed lower limit	No prescribed lower limit
Ni (mg L^−1^)	0.2631	3	No prescribed lower limit	No prescribed lower limit
Pb (mg L^−1^)	Not detected	0.1	No prescribed lower limit	No prescribed lower limit
Na (mg L^−1^)	42.0972	No prescribed lower limit	No prescribed lower limit	No prescribed lower limit
Mn (mg L^−1^)	0.0105	No prescribed lower limit	No prescribed lower limit	No prescribed lower limit
Ca (mg L^−1^)	289.7853	No prescribed lower limit	No prescribed lower limit	No prescribed lower limit
K (mg L^−1^)	Not detected	No prescribed lower limit	No prescribed lower limit	No prescribed lower limit
Mg (mg L^−1^)	10.374	No prescribed lower limit	No prescribed lower limit	No prescribed lower limit
N (mg L^−1^)	3.5	No prescribed lower limit	No prescribed lower limit	No prescribed lower limit

### Isolation and identification of native algal strain present in oil field formation water

**Figure 1:**
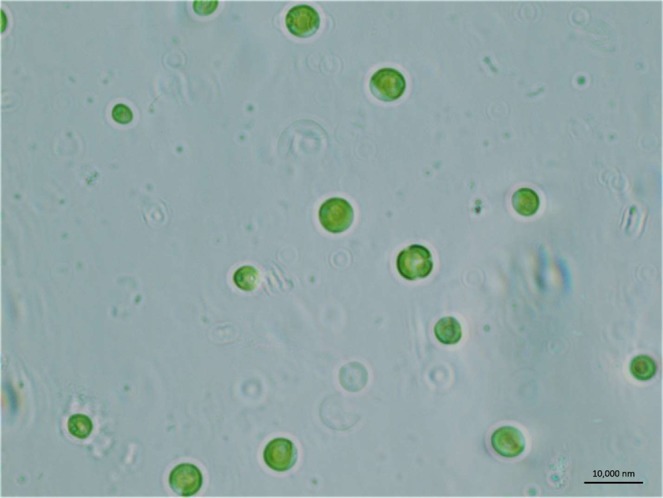
Optical microscope image of *Chlorella vulgaris* BS1 at 100X magnification.

We isolated a native algal strain of *Chlorella vulgaris* BS1 present in oil field formation water. The morphological characteristics of the algal isolate BS1 (Fig. 1) is unicellular (rarely aggregated into small groups), spherical, green color with no extension of flagella^16–19^. These morphological characteristics indicated the algal isolate initially named as Sample P3 to be *Chlorella* species. The morphological identification is followed by 18S ribosomal RNA (18S rRNA) based identification of the algal isolate Sample P3. The first ten sequences selected based on maximum identity score obtained from BLAST analysis of the 18S rRNA sequence of the algal isolate were: *Chlorella vulgaris* genomic DNA containing 18S rRNA gene (Max Score: 1901; NCBI Accession No: FR865683.1), *Chlorella chlorelloides* strain CB 2008/110 18S ribosomal RNA gene (Max Score: 1901; NCBI Accession No: HQ111432.1); *Heynigia riparia* strain CCAP 222/47 18S ribosomal RNA gene (Max Score: 1899; NCBI Accession No: GQ487225.1); *Hindakia fallax* strain CCAP 222/29 18S ribosomal RNA gene (Max Score: 1899; NCBI Accession No: GQ487223.1); *Pseudochlorella pringsheimii* 18S ribosomal RNA gene (Max Score: 1895; NCBI Accession No: KY364701.1); *Chlorella sorokiniana* strain KLL-G018 clone a 18S ribosomal RNA gene (Max Score: 1895; NCBI Accession No: KP726221.1), *Chlorella sorokiniana* 18S rRNA gene (Max Score: 1895; NCBI Accession No: LK021940.1), *Chlorella singularis* strain CB 2008/73 18S ribosomal RNA gene (Max Score: 1895; NCBI Accession No: HQ111435.1), *Chlorella volutis* strain CB 2008/69 18S ribosomal RNA gene (Max Score:1895; NCBI Accession No: HQ111434.1), *Hindakia tetrachotoma* strain CCAP 222/69 18S ribosomal RNA gene (Max Score:1895; NCBI Accession No: GQ867590.1). These sequences were aligned using multiple alignment software program Clustal W followed by generation of the distance matrix to estimate the evolutionary divergence between the sequences. The evolutionary history was inferred by using the Maximum Likelihood method based on the Kimura 2-parameter model. The number of base substitutions per site from between sequences are shown in the distance matrix with standard error estimate(s) shown above the diagonal (Supplementary Table 1). The distance matrix analysis involved 11 nucleotide sequences. The codon positions included were 1st + 2nd + 3rd + Noncoding. From the distance matrix (Supplementary Table 1), the 18S rRNA sequence of the algal isolate (Sample P3) was found to have least evolutionary divergence with *Chlorella vulgaris* (NCBI Accession No: FR865683.1). The nucleotide blast result also supported that the algal isolate is *Chlorella vulgaris* by providing max score of 1901, 99% query coverage, E value of 0, 99% sequence identity with *Chlorella vulgaris* (NCBI Accession No: FR865683.1). Thus, on basis of the results from the phylogenetic analysis (based on Maximum Likelihood method and Kimura 2-parameter model) and the distance matrix generated, it showed that the algal isolate is *Chlorella vulgaris* and hence named as *Chlorella vulgaris* BS1. The 18S rRNA gene sequence of *Chlorella vulgaris* BS1 was deposited to NCBI GenBank, and an Accession No. MH732950 was obtained. The main bottleneck behind an efficient bioremediation process is to select microalgal species that is a good fit for cultivation in the oil field formation water. In this context, the algal isolate being native to the oil field formation water could be beneficial since it is already adapted to grow in oil field formation water, which is indispensible for developing an efficient remediation process.

### Growth kinetics of the algal isolate in oil field formation water

**Figure 2:**
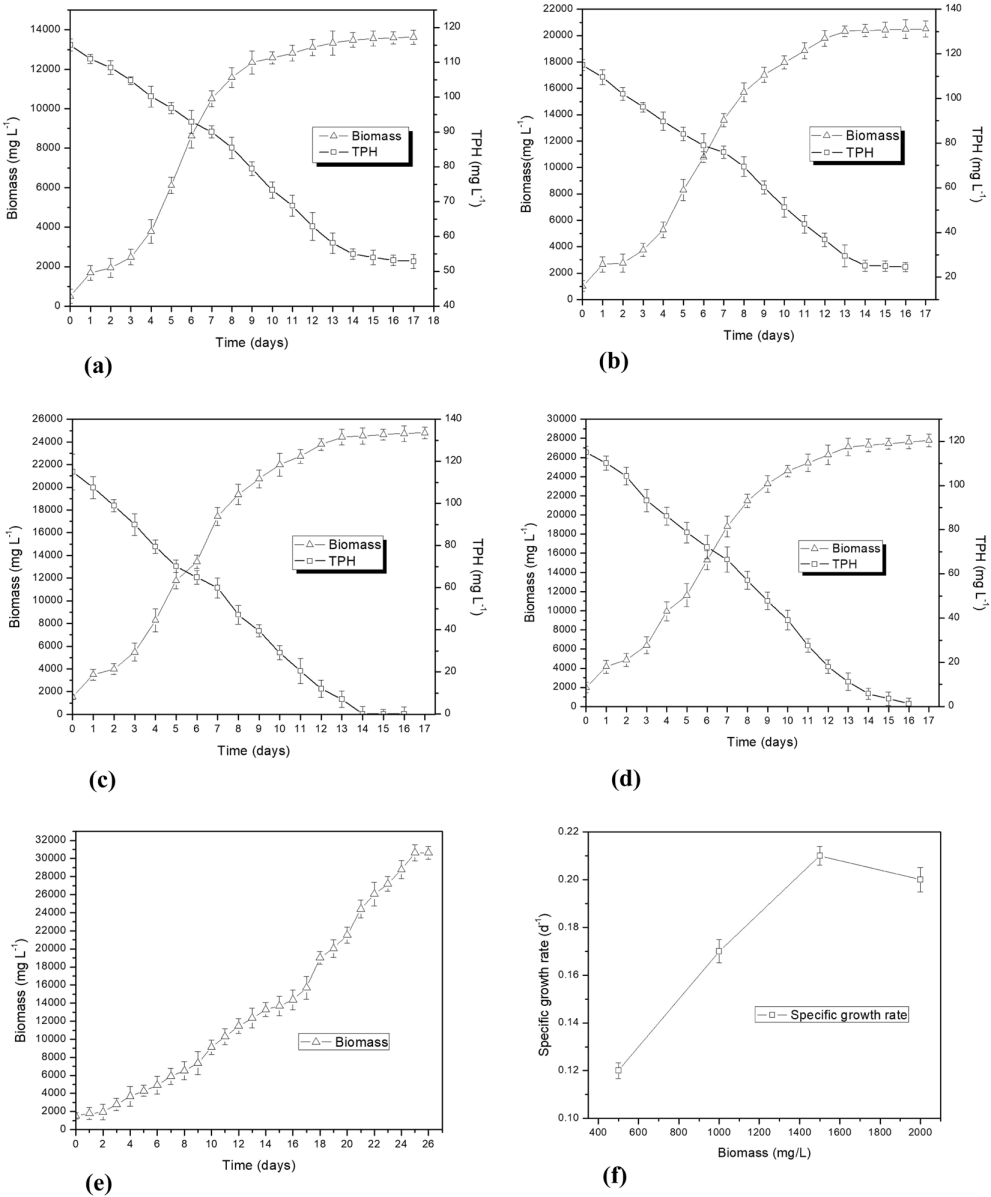
Biomass growth and TPH degradation profile by *Chlorella vulgaris* BS1 in oil field formation water at various inoculum concentrations: (**a**) 500 mg L^−1^ (**b**) 1000 mg L^−1^ (**c**) 1500 mg L^−1^ (**d**) 2000 mg L^−1^ (**e**) Control; (**f**) Specific growth rate of various inoculum concentrations (500–2000 mg L^−1^) of *Chlorella vulgaris* BS1 in oil field formation water.

The rate of biodegradation is a strong function of biomass growth rate. Thus, any medium where the microbes can grow faster will result in better biodegradation rate^20^. Thus, an understanding of the growth kinetics of the algal isolate *Chlorella vulgaris* BS1 in oil field formation water is required to realize its potential for efficient hydrocarbon remediation. The efficiency of microalgal remediation system is dependent on inoculum dose. The application of high concentration of algal biomass could cause desired remediation to be achieved within shorter incubation time. However, a high microalgal biomass load could result in self-shading which could compromise algal growth and hence the remediation efficiency^21^. The present study explores the growth kinetics of *Chlorella vulgaris* BS1 in oil field formation water with different inoculum doses in the range of 500–2000 mg L^−1^. The growth of Chlorella vulgaris BS1 in relation with TPH degradation in formation water has been shown for various initial inoculum (500–2000 mg L^−1^) of the algal strain in Fig. 2a–d respectively. Along with this, Fig. 2f depicts the specific growth rate attained by various inoculum (500–2000 mg L^−1^) of *Chlorella vulgaris* BS1 in oil field formation water. Among the different inoculums concentrations tested, the specific growth rate increased with increase in inoculums concentration until the highest specific growth rate of 0.21 d^−1^ was obtained at 1500 mg L^−1^ inoculum (Fig. 2f). However, the specific growth rate decreases with increase in inoculum concentration beyond 1500 mg L^−1^. Das *et al*.^21^ while studying phenol degradation by *Chlorella pyrenoidosa* reported similar phenomena of increased specific growth rate with the increase in inoculum concentration until a maximum inoculum concentration of 200 mg L^−1^ beyond which the specific growth rate declines. For control cultures (Fig. 2e), the specific growth rate of 0.12 d^−1^ is lower compared to that of 0.21 d^−1^ obtained in oil field formation water (statistically significant, p < 0.05). Consequently, the biomass productivity of 1.76 gm L^−1^ d^−1^ achieved by *Chlorella vulgaris* BS1 cultivated in oil field formation water is higher compared to that of 1.25 gm L^−1^ d^−1^ obtained for control cultures (statistically significant, p < 0.05). The chlorophyll A content of *Chlorella vulgaris* BS1 cultivated in oil field formation water for 14 days is 54.49% higher compared to that in control cultures which further supports the high biomass growth rate of *Chlorella vulgaris* BS1 in formation water (Supplementary Fig. 1). Talebi *et al*.^11^ cultivated *Dunaliella salina* in various dilutions of oil field produced water and sea water as 1:1, 1:2, 1:3 and sea water (control) with biomass productivity of 2.75 gm L^−1^, 2.50 gm L^−1^, 1.75 gm L^−1^ and 1.25 gm L^−1^ respectively after 25 days of incubation. Similarly, *N*. *salina* CCMP 1776 cultivated in oil field produced water reported biomass productivity of 2.7 gm L^−1^ after 24 days of incubation^22^. The present study achieved higher biomass production of 24.53 gm L^−1^ in oil field formation water within a shorter incubation time of 14 days with no requirement of dilution or additional nutrient supplementation which makes *C*. *vulgaris* BS1 more efficient in remediation method.

**Figure 3:**
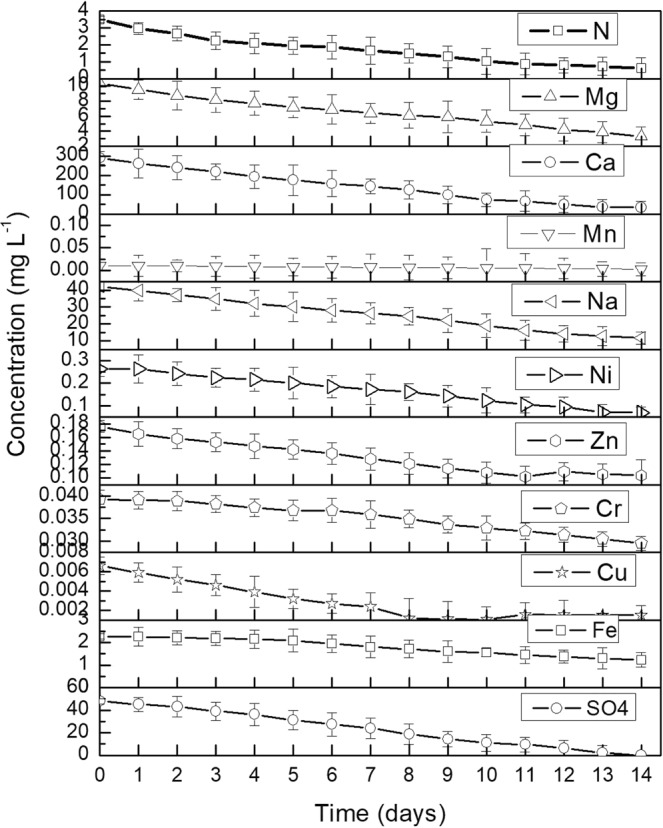
Uptake profile of N, Mg, Ca, Mn, Na, Ni, Zn, Cr, Cu, Fe and SO_4_ by *Chlorella vulgaris* BS1 during its growth in oil field formation water.

The inorganic composition of oil field formation water contains a range of major constituents which are essential for algal media creation and cultivation. Oil field formation water contains sodium, chloride and sulfate as the major ions, all of which have nutritive importance for algal cultivation. Some other constituents of oil field formation water as iron, copper, manganese and zinc are important micronutrients quintessential for algal growth. This indicates the possibility to utilize oil field formation water as a source of algal cultivation media nutrients^22^. As discussed above (Table 1), the oil field formation water contains several major constituents as SO_4_, Fe, Cu, Zn, Na, Mn, Ca, N and Mg which are essential constituents of standard algae cultivation media like Fog’s media commonly used to cultivate *Chlorella* species. Figure 3 and Table 2 represents how the inorganic components present in the oil field formation water were consumed by the algal isolate *Chlorella vulgaris* BS1 during its growth and concomitant TPH removal from oil field formation water (the biomass growth and TPH degradation profile is represented in Fig. 2c) resulting in no requirement of additional nutrients for TPH removal from formation water. A prominent decrease in concentrations of the inorganic components (commonly present in standard algal cultivation media) as SO_4_ (100%), Fe (45.84%), Cu (77.27%), Cr (24.94%), Zn (41.18%), Na (72.1%), Ca (88.48%), Mn (75.24%), Mg (67.9%) and N (82.57%) in the formation water sample following cultivation of *Chlorella vulgaris* BS1 indicates its utilization as growth nutrients (Fig. 3 and Table 2). The oil field formation water typically contains high concentrations of sulfate in the range of 2 to 1650 mg L^−1^ ^15,23^. In accordance with this, the oil field formation water was found to be sulfate-rich containing 48 mg L^−1^ sulfate (Table 1). Sulfur is an important macronutrient for microalgal growth with its biomass content ranging from 0.15% to 1.6%. Sulfur is a key component of the amino acids cysteine and methionine; sulfolipids in cell membranes; oligopeptides (glutathione and phytochelatins); vitamins and cofactors (biotin, thiamine, CoA and S-adenosyl-Met); regulatory compounds as well as sulfur-containing secondary metabolites in microalgae^24,25^. The sulfur requirements of microalgae are fulfilled mainly in form of sulfate. The sulfate is absorbed by cells and reduced in the plastids for the formation of cysteine and methionine and various sulfur-containing coenzymes as well as metabolites^25^. In accordance with this, *Chlorella vulgaris* BS1 could completely utilize sulfate present in formation water within 14 days of incubation (Fig. 3 and Table 2). In accordance with this, the algal strain could utilize 34.18% sulfate present in control cultures over the same incubation period of 14 days (Supplementary Fig. 3). The sulfate-rich formation water presents a challenge for its reuse as injection water for oil drilling activities. The high concentration of sulfate in formation water results in stimulating the growth of sulfate-reducing bacteria in the oil reservoirs subsequently resulting in H_2_S formation. This process of biogenic H_2_S production known as “reservoir souring” is a cause of grave concern to the oil industry owing to reservoir plugging, toxic and corrosive nature of H_2_S and increased content of sulfur in oil and gas. In order to prevent the production of H_2_S, biocides as glutaraldehyde are incorporated into injection water. However, the biocide treatment suffers from drawbacks as being expensive along with its reduced effectiveness owing to the growth of sulfate-reducing bacteria in biofilms and biocide inactivation after reaction with biofilm as well as minerals. The use of recalcitrant biocides is another matter of environmental concern while decomposition of biocides may yield substrates for sulfate-reducing bacteria. Nanofiltration to remove sulfate is efficient but is very costly for large-scale application. The need of the hour is to devise a cost-effective biological process for remediation of sulfate present in the formation water prior to its reinjection for oil drilling^26^. As discussed earlier, the ability of *Chlorella vulgaris* BS1 to completely utilize sulfate anion as a sulfur source indicates its promising ability to be used for removal of sulfate present in oil field formation water. Mohammadi *et al*.^25^ evaluated the ability of five microalgal species (*Chlorella* sp., *Chlamydomonas* sp., *Oocystis* sp., *Scenedesmus* sp., and *Fischerella* sp.) to remediate sulfate from power plant wastewater in the batch culture system. They reported that the microalgal species could efficiently remove 22–32% sulfate within 21 days of incubation which supports the findings of the present study. Bako *et al*.^27^ also reported similar findings of 25.56% sulphate removal along with remediation of hydrocarbons in refinery effluents by a consortium of *Pseudomonas aeruginosa* and *Penicillium janthinellum* following an incubation period of 2 weeks. However, they supplemented the refinery effluent with nutrients to maintain the growth requirements of bacterial consortia. The present study reports 100% sulfate remediation present in oil field formation water without any requirement of nutrient supplementation. This adds to practical applicability of the strain for cost-effective and faster treatment of sulfate-rich formation water making it suitable for reinjection into the oil reservoirs. The priority pollutant Ni, present in formation water sample can be utilized as growth nutrient by some algal species^22^. In accordance with this, the algal isolate *Chlorella vulgaris* BS1 reported an uptake of 73.51% of Ni during its cultivation in formation water indicating its possible utilization as a micronutrient for growth (Table 2). The ability of microalgal species to grow in presence of toxic metals as Ni is contributed by its capacity for adsorption of these ions and the role these ions play as cofactors for metalloenzymes^28^. Lustigman *et al*.^28^ studied the effect of Ni on growth of *Chlorella vulgaris*. They concluded that the growth of *Chlorella vulgaris* increases in presence of 10 ppm Ni which supports the findings of the present study. Thus, *Chlorella vulgaris* BS1 could achieve the high biomass productivity in oil field formation water by virtue of its algal growth feasible ionic composition. This overcomes the requirement of nutrient supplementation of oil field formation water to maintain the growth requirements of the algal isolate.

**Table 2:** Characteristics of oil field formation water after treatment with *Chlorella vulgaris* BS1 for 14 days and its compliance with environmental standards for its disposal and reuse.

Characteristics of oil field Formation water after treatment	Values	Does the treated formation water satisfy the regulatory guidelines for its disposal or reuse?		
		On-shore disposal (Environment Protection Rules, 1986)	Disposal by reinjection into abandoned oil well (Environment Protection Rules, 1986)	Reuse by reinjection into oil reservoirs (Recommendation Standard and Analysis Method for the Clastic Rock Reservoir Injection Water Quality)
pH	7.2 ± 0.18	✓	No regulatory limits	No regulatory limits
TPH (mg L^−1^)	0.04 ± 0.12	✓	✓	✓
SO_4_ (mg L^−1^)	Not detected	✓	No regulatory limits	✓
COD (mg L^−1^)	79.2 ± 2.61	✓	No regulatory limits	No regulatory limits
Fe (mg L^−1^)	1.232 ± 1.03	✓	No regulatory limits	No regulatory limits
Cu (mg L^−1^)	0.0015 ± 1.02	✓	No regulatory limits	No regulatory limits
Cr (mg L^−1^)	0.0295 ± 0.03	✓	No regulatory limits	No regulatory limits
Zn (mg L^−1^)	0.1037 ± 0.001	✓	No regulatory limits	No regulatory limits
Ni (mg L^−1^)	0.0697 ± 0.004	✓	No regulatory limits	No regulatory limits
Na (mg L^−1^)	11.744 ± 1.21	No regulatory limits	No regulatory limits	No regulatory limits
Mn (mg L^−1^)	0.0026 ± 1.06	No regulatory limits	No regulatory limits	No regulatory limits
Ca (mg L^−1^)	33.376 ± 0.17	No regulatory limits	No regulatory limits	No regulatory limits
Mg (mg L^−1^)	3.3298 ± 0.013	No regulatory limits	No regulatory limits	No regulatory limits
N (mg L^−1^)	0.61 ± 0.04	No regulatory limits	No regulatory limits	No regulatory limits

### Remediation of oil field formation water

**Figure 4:**
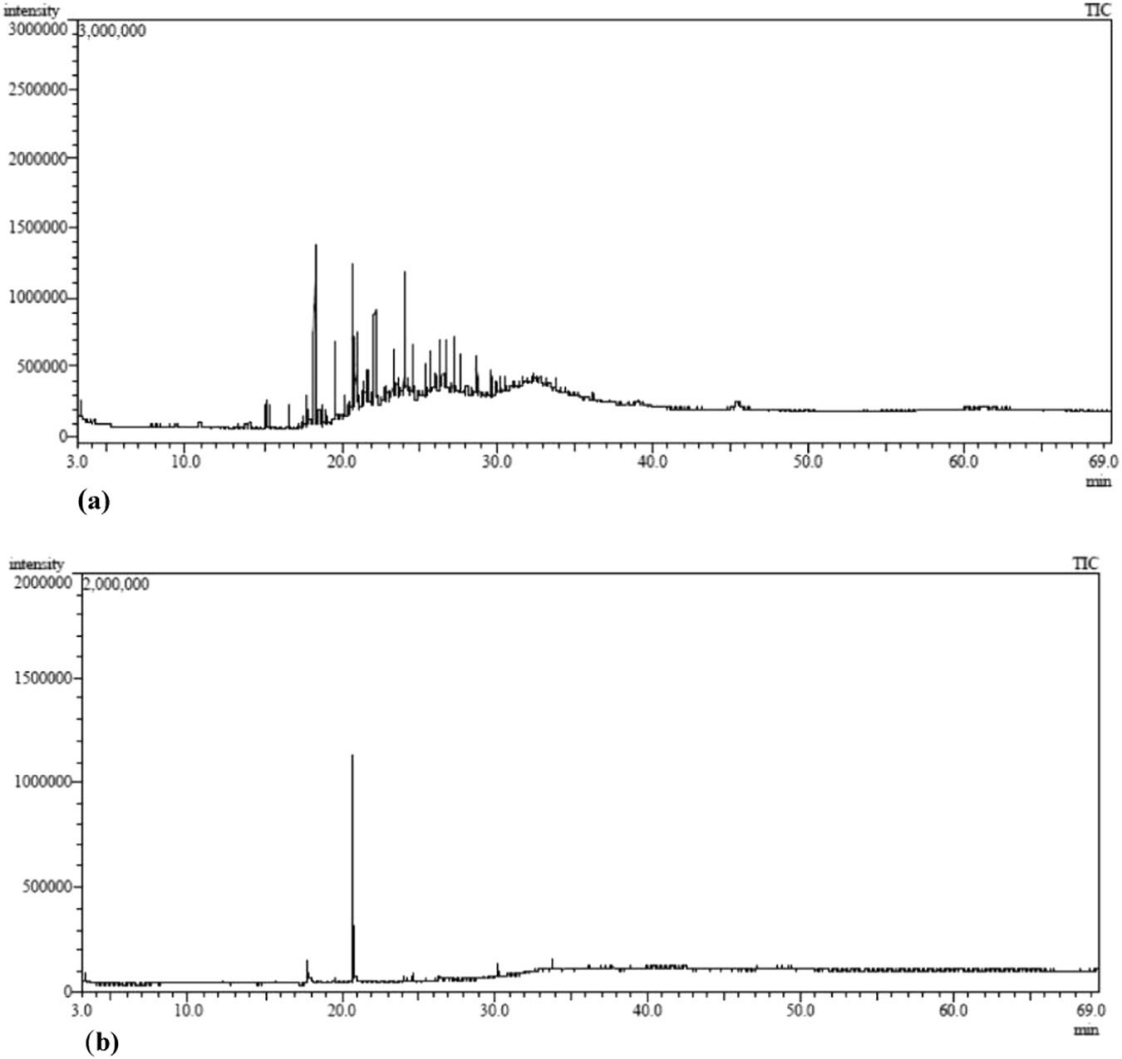
(**a**) Gas chromatography-mass spectrometer (GC-MS) chromatograph of abiotic control formation water incubated for 14 days. (**b**) GC-MS chromatograph of formation water treated with *Chlorella vulgaris* BS1 for 14 days.

**Figure 5:**
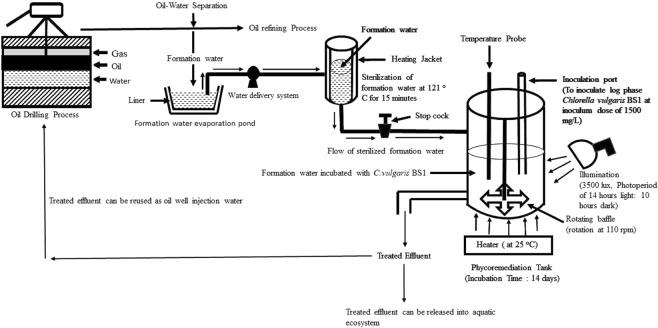
Schematic diagram of the proposed phycoremediation process for treatment of oil field formation water.

Considering the virtues of the algal isolate *Chlorella vulgaris* BS1 of high biomass productivity (1.76 gm L^−1^ d^−1^) with no requirement of external nutrient supplementation or dilution of oil field formation water brightens its potential to find a practical application for remediation of oil field formation water. *Chlorella* sp. could efficiently utilize petroleum hydrocarbons as carbon source through its mixotrophy^20,21^. Considering this, the algal isolate *Chlorella vulgaris* BS1 could grow by utilizing the hydrocarbons present in oil field formation water as a source of carbon. The analysis of TPH degradation in formation water by *Chlorella vulgaris* BS1 in inoculum range of 500–2000 mg L^−1^ indicates that the highest increment in degradation (%) in successive interval of 7 days and consequently the highest TPH degradation of 98.63% (after incubation of 14 days) was obtained with 1500 mg L^−1^ inoculum (Fig. 2c and Supplementary Table 2). This may be attributed to highest specific growth rate in formation water obtained with 1500 mg L^−1^ inoculum of *Chlorella vulgaris* BS1 (Fig. 2f). Das *et al*.^21^ reported similar phenomena while studying phenol degradation by *Chlorella pyrenoidosa*. They reported that the highest phenol degradation was achieved at the algal inoculum where the highest specific growth rate was obtained which supports our present findings. The residual hydrocarbon profile of the 1500 mg L^−1^ inoculum culture which showed highest TPH degradation was analyzed by GC-MS and described in Fig. 4b. The comparison of the residual hydrocarbon profile (Fig. 4b) with that of abiotic control (Fig. 4a) indicated that *Chlorella vulgaris* BS1 could completely degrade a range of hydrocarbonaceous compounds (C12 to C60) comprising of alkanes and polyaromatic hydrocarbon (PAH) present in formation water (Supplementary Table 3). The GC-MS results validate the findings of gravimetric analysis that 98.63% TPH was degraded by *Chlorella vulgaris* BS1 within 14 days of incubation. The potential of microalgae to degrade petroleum hydrocarbons has been reported by a number of previous studies. Kalhor *et al*.^29^ reported that *Chlorella vulgaris* could remediate 88% of crude oil hydrocarbon compounds within an incubation time of 14 days which supports our present findings. However, it is to be noted that the hydrocarbon degradation was studied in Konvey medium for maintenance of algal growth requirements. Similarly, the reports related to the ability of green microalgal species *S*. *obliquus* and *C*. *vulgaris* to degrade and utilize crude oil hydrocarbons as a carbon source^30^ is in accordance with findings of the present study. El-Sheekh and Hamouda^31^ reported the ability of *Nostoc punctiforme* and *Spirulina platensis* to utilize aliphatic compounds present in crude oil as carbon source which corroborates to the results of the present study. However, it is to be noted that all of the above-mentioned hydrocarbon degradation experiments were carried out in standard algal media for maintenance of the growth requirements of algal biomass. By far, when algae was applied for phycoremediation of real wastewater as petroleum hydrocarbon rich formation water it did not exhibit the desired hydrocarbon removal efficiency. Talebi *et al*.^11^ evaluated the application of algal strain *Dunaliella salina* to remediate diluted oil field formation water. They reported that *Dunaliella salina* could not degrade TPH present in the diluted produced water sample. Apart from algal bioremediation, the microbial treatment of formation water has been reported by a number of previous studies. Sharghi *et al*.^32^ studied the biological treatment of synthetic formation water by mixed bacterial culture in a membrane bioreactor. The synthetic formation water used contained all mineral salt medium according to the growth requirements of the mixed bacterial structure. It was reported that the bacterial consortium could degrade most of the hydrocarbon fractions in synthetic formation water except the high molecular weight compounds following 106 days of operation of the reactor. Similarly, Habibi and Babaei^33^ analyzed the degradation of hydrocarbons in oil-field formation water by *Candida catenulata*. They found that 95% TPH degradation by *Candida catenulata* is possible only after supplementation of the oil field formation water with nutrient requirements of the yeast cells. On the same lines, Tellez *et al*.^7^ evaluated the degradation of petroleum hydrocarbons in formation water generated by the southwestern US oilfield by an activated sludge treatment unit. The formation water was supplemented with macronutrients for appropriate microbial growth. They reported TPH removal efficiency of 98–99% at a solids retention time of 20 days. In contrary to the previous studies, the present study achieved 98.63% remediation of TPH in oil field formation water without any requirement of nutrient supplementation or dilution of the oil field formation water. This adds to practical applicability of *Chlorella vulgaris* BS1 for cost-effective and efficient remediation of petroleum hydrocarbons in formation water overcoming the bottlenecks as requirements of external growth nutrient addition and dilution associated with conventional biological treatment. The *in-situ* biological treatment of hazardous organic pollutants require injection of microrganisms along with nutrients for maintaining the biomass growth. The reduction of cost associated with this requirement of inorganic nutrients can go a long way to make biological treatment cost-effective and sustainable. In this context, the present phycoremediation process cuts down costs associated with supply of nutrients. This is evident from the cost-wise calculation associated with nutrient requirement as mentioned in Table 3. As evident from Table 3, the cost of nutrients supply for 1000 L control culture is INR 6114 (89.18 US Dollars). On the other hand, the present phycoremediation approach overcomes the requirements of nutrient supplementation to maintain biomass growth and hence results in a cost-effective remediation process (Table 3). The present process with no requirement of nutrient supply, could sustainably produce significantly high biomass productivity of 1.76 gm L^−1^ d^−1^ as against that in control (1.25 gm L^−1^ d^−1^) which is indispensable for efficient remediation. Figure 5 represents a schematic diagram of the proposed phycoremediation process for oil field formation water. As schematically represented in Fig. 5, a specified volume of formation water to be treated is transferred from open evaporation ponds which stores formation water for natural evaporative treatment. A water delivery system transfers this required volume of formation water from evaporation ponds into sterilization chamber where it is subjected to sterilization at 121 °C for 15 minutes. Then, the sterilized formation water is flown to into a phycoremediation tank made of transparent glass for light supply. In the phycoremediation tank, the formation water is inoculated with the algal strain *Chlorella vulgaris* BS1 at inoculum concentration of 1500 mg/l. The algal biomass is cultivated in the phycoremediation tank under the cultivation conditions as illumination of 3500 lux for a photoperiod of 14 hours light: 10 hours dark, mixing of the culture at 110 rpm by a rotating baffle and incubation temperature of 25 °C. For phycoremediation of the formation water, the phycoremediation tank is operated at the above mentioned cultivation conditions for a time period of 14 days. After the incubation period of 14 days, the treated effluent achieved could be safely discharged or be reused as oil well injection water. The formation water used for the present process is obtained from open evaporation ponds which are routinely employed for on site or off site treatment of formation water by evaporating the water by solar energy. However, the use of evaporative ponds for formation water is not applicable when water recovery for reuse is an objective of the water treatment strategies. In this context, the present phycoremediation process could serve as a sustainable approach by recovering a portion of the water resource from formation water evaporation ponds which would otherwise be lost to the environment.

**Table 3:** Comparison of cost of nutrient requirements and biomass growth in oil field formation water and in control.

Medium	Biomass productivity (gm L^−1^ d^−1^)	Specific growth rate (d^−1^)	Cultivation time (days)	Cost of nutrients (US dollars/1000 L)
Oil field formation water	1.76	0.21	13	—
Control	1.25	0.12	25	89.18

*Chlorella vulgaris* BS1 in the present study efficiently undergo mixotrophic growth using both hydrocarbons as well as inorganic carbon through photosynthesis as carbon source. In order to verify the role of mixotrophic growth and hence the photodependency of the present phycoremediation process, the incubation of *C*. *vulgaris* BS1 in oil field formation water was carried out in dark condition where hydrocarbons in formation water is the only carbon source. Supplementary Fig. 2 depicts the relationship between biomass growth and TPH degradation when *Chlorella vulgaris* BS1 was incubated in dark. It has been found that algal growth has been significantly reduced in dark as evidenced by lower specific growth rate (0.066 d^−1^) and biomass productivity (0.12 g L^−1^ d^−1^) as compared to that in case of mixotrophic growth conditions provided in the present study (specific growth rate: 0.21 d^−1^; biomass productivity: 1.76 g L^−1^ d^−1^). In relation with the lower biomass growth in dark cultures, the degradation of TPH present in formation water also significantly decrease with only 11.45% degradation within 7 days incubation as against 47.89% TPH removal within the same incubation period under mixotrophic cultivation conditions described in present study. Das *et al*.^20^ compared the biomass growth and phenol utilization by *Chlorella* sp. under mixotrophic cultivation condition and in dark. They reported negligible biomass growth and phenol degradation in dark as compared to that in the mixotrophic process indicating photodependency of the process which corroborates to findings of the present study.

Biodegradation of xenobiotic compounds is characterized by the accumulation of breakdown metabolites as well as new metabolites produced in the fermentation media. The GC-MS chromatogram of the treated formation water (Fig. 4b) indicates the presence of the degradation intermediates as primary alcohols namely 1-undecanol (Retention time: 17.740 min) and n-tridecan-1-ol (Retention Time: 20.739 min) as well as degradation intermediates forming acids as propanoic acid (Retention Time: 20.833 min), decanoic acid (Retention time: 26.414 min), dodecanoic acid (Retention time: 30.255 min) and hexadecanoic acid (Retention time: 33.762 min), all of which are commonly reported petroleum hydrocarbon breakdown products^34–40^. The degradation intermediate n-tridecan-1-ol (C_13_H_28_O; Retention Time: 20.739 min) shows the highest intensity in the GC-MS chromatogram which is a long chain fatty alcohol with lower solubility in water as compared to its short chain homologues. This low water solubility does not allow long chain fatty alcohols to penetrate the cytoplasmic membrane in high concentrations. On the other hand, the shorter-chain fatty alcohols by virtue of its high solubility in water could penetrate the cytoplasmic membrane in relatively high concentrations resulting in disturbances in membrane permeability as well as inhibition of endogenous respiration^41^. Hence, the accumulation of long chain fatty alcohol n-tridecan-1-ol as hydrocarbon breakdown metabolite is least likely to compromise the efficiency of the phytoremediation process. Aerobic alkane-degrading microbes activate alkane molecules using O_2_ as a reactant. The alkane-activating monooxygenase counters the hydrocarbons with low reactivity by the production of reactive oxygen species. The alkane degradation is initiated by oxidation of terminal methyl group to primary alcohol followed by its oxidation to aldehyde and finally to a fatty acid. In the case of aromatics, the microbial biodegradation pathways result in the conversion of the aromatic compounds to ortho or paradihydroxy phenol derivatives followed by cleavage of the ring to aliphatic acids. Finally, these are processed by the β-oxidation pathway^42,36^. The accumulation of the above mentioned TPH breakdown metabolites in the present study indicates the role of enzymatic pathways in conferring petroleum hydrocarbon degradation ability to *Chlorella vulgaris* BS1. Supplementary Fig. 4 represents the amount of TPH dissipated after 1 day of incubation with dead biomass (sorption) which will help verify if TPH dissipation is mainly contributed by sorption or biodegradation. Following incubation for 1 day, the dead biomass dissipated only 2.5% TPH from formation water contributed by sorption process. On the contrary, live cells could degrade significantly higher 6.52% TPH over the same incubation time period (Fig. 2c). Hence, on basis of accumulation of biodegradation intermediates following formation water treatment and low TPH removal by sorption, the dissipation of TPH by the algal strain is mainly due to biodegradation and not related only to sorption to algal biomass.

The petroleum hydrocarbons are the major contributor to COD of oil field formation water. In accordance with this, the COD in the formation water sample is well beyond the permissible limit prescribed by regulatory agencies for its safe disposal (Table 1). In order to dispose of the formation water, it has to compile with the environmental regulatory standards of COD. In this context, strategy to exploit the ability of microbes to degrade petroleum hydrocarbons in formation water generates significant interest. However, conventional biological treatment does not exhibit efficient performance at hypersaline conditions of formation water^43^. Considering the indigenous nature of the algal isolate *Chlorella vulgaris* BS1 to oil field formation water along with its potential of high biomass productivity (1.76 gm L^−1^ d^−1^) and efficient hydrocarbon remediation (98.63% TPH removal) in oil field formation water generated interest to evaluate the efficiency of the algal isolate for reduction of COD load in oil field formation water. *Chlorella vulgaris* BS1 resulted in 75% decrease in COD load present in the formation water sample conforming it to environmentally permissible disposal limits (Table 2). Gupta *et al*.^44^ studied the reduction of COD at different dilutions of synthetic food processing industry wastewater by microalgal species *Chlorella pyrenoidosa*. They reported a significantly high COD reduction of 61–66% by the microalgal strain in diluted wastewater as compared to low COD reduction of 43% in non-diluted wastewater. Madadi *et al*.^45^ analyzed the applicability of *Chlorella vulgaris* for remediation of 20% diluted petrochemical wastewater and reported 11.33% COD removal. However, the supplementation of the process with commercial surfactants resulted in an increase in COD removal rates to 15–38%. In contrary to the previous studies, the significant reduction of 75% COD load by the algal isolate *Chlorella vulgaris* BS1 without any requirement of freshwater for dilution of the oil field formation water is of significant advantage for realizing a sustainable and efficient remediation process.

Phototrophic organisms as algae owing to its capability of photosynthesis has high intracellular oxygen content. When algal cells are subjected to salinity stress, Reactive Oxygen Species (ROS) as superoxide radicals, singlet oxygen, hydrogen peroxide and hydroxyl radical are formed which could result in damage of biomacromolecules as DNA, lipids and proteins. The protection against potential damage by ROS is provided by intracellular antioxidants as carotenoids which quenches the ROS and hence makes it possible for the algal cells to maintain its biomass growth in saline environments^46,47^. The intracellular production of ROS in algae subjected to salinity stress, dramatically induces expression of crt genes resulting in enhanced biosynthesis of carotenoids^46^. In accordance with this, *Chlorella vulgaris* BS1 cultivated in oil field formation water with a salinity of 2.24 PSU (Practical Salinity Unit) showed 2.22 fold enhancement in accumulation of carotenoids (142 µg/gm) as compared to that of 64 µg/gm in case of control cultures (statistically significant, p < 0.05). This indicates that the algal isolate *Chlorella vulgaris* BS1 involves a mechanism for adaptation to saline stress that correlates with enhanced carotenoid biosynthesis. This finding of enhanced carotenoid production as a strategy to counter salinity stress corroborates with similar results of high carotenoid accumulation in *Dunaliella* salina^47,48^^,^; *Scytonema javanicum*^49^; *Spirulina platensis*^50^; *Botryococcus braunii*^51^ in response to salinity. The intracellular antioxidant mechanism enables *Chlorella vulgaris* BS1 to efficiently tolerate salinity stress as evident from the high biomass productivity of 1.76 gm L^−1^ d^−1^ achieved by algal strain cultivated in formation water as compared to 1.25 gm L^−1^ d^−1^ achieved for control cultures (statistically significant, p < 0.05). This ability of the *Chlorella vulgaris* BS1 to maintain high productivity of biomass by withstanding salinity stress in formation water results in an efficient phycoremediation process with decrease in salinity (50%), TDS (51.46%), TSS (11.98%), TOC (88.27%) along with a decrease in Sodium Adsorption Ratio (SAR) value from 0.66 to 0.52 (Table 4). The TDS content represents the salinity hazard of water sample^52^. The 51.46% TDS removal by the algal treatment decreased the TDS concentration in the treated formation water significantly below the permissible discharge limit of 2000 mg L^−1^ as prescribed for oil industry wastewaters in The Environment (Protection) Rules, 1986 of Govt. of India^53^.

**Table 4:** Variation in formation water quality parameters before and after phycoremediation.

Parameters	Before treatment	After treatment
Salinity (PSU)	2.24 ± 0.21	1.12 ± 0.18
TDS (mg L^−1^)	2365 ± 0.78	1148 ± 1.03
TSS (mg L^−1^)	38.7 ± 1.05	26.72 ± 1.97
TOC (mg L^−1^)	185.27 ± 0.05	21.73 ± 1.12
SAR	0.66 ± 0.43	0.52 ± 0.57

## Conclusion

The present study developed a new method for remediation of petroleum hydrocarbon-rich formation water. Since the microalgal remediation process involved the application of algal species native to oil field formation water, the algal isolate *Chlorella vulgaris* BS1 could meet its growth requirement of carbon and macro/micro-nutrients from the petroleum hydrocarbons and ionic composition of formation water respectively. Owing to this, *Chlorella vulgaris* BS1 achieved significantly high biomass productivity of 1.76 gm L^−1^ d^−1^ (specific growth rate: 0.21 d^−1^, Initial inoculum: 1500 mg L^−1^) in oil field formation water without any requirement of dilution or additional nutrient supplementation which is significantly advantageous to develop a practically applicable and efficient remediation process. In accordance with this, the present process resulted in 98.63% remediation of TPH in oil field formation water with very minimal accumulation of breakdown metabolites indicating an efficient hydrocarbon remediation process. The microalgal remediation process resulted in 75% reduction of COD load in the formation water meeting the environmentally permissible disposal standards. Concomitantly, *Chlorella vulgaris* BS1 could completely remediate sulfate present in oil field formation water which is quintessential to realize its reuse as oil well injection water. Thus, the present process overcoming the bottlenecks of external growth nutrient addition or dilution associated with conventional biological treatment results into a practically applicable and cost-effective technology for remediation of oil field formation water for its safe disposal or reuse.

## Methods

### Formation water sample and chemicals

Formation water samples for the experiments were collected in sterile sample bottles from oil field of Oil and Natural Gas Corporation (ONGC) in upper Assam (26.98°N 94.63°E), India. The samples were stored in ice packs while transferring it to the laboratory followed by its storage at 4 °C until further analysis. All the chemicals used in the experiments were obtained from Merck, India.

### Characterization of the formation water sample

#### Analysis of total petroleum hydrocarbon (TPH) in formation water

The TPH present in the formation water was extracted by solvent extraction method using dichloromethane (DCM). This is followed by evaporation of the solvent using a rotary evaporator (Rotavapor R-210, Buchi, Switzerland), transferring the hydrocarbon solution to a pre-weighed beaker and dried in room temperature until a constant weight is acheived. The gravimetric analysis of TPH in formation water was carried out by measuring the difference in weight between the pre-weighed beaker and the dried hydrocarbon containing beaker^54^. Apart from this, the extracted hydrocarbon was analyzed by a triple quadruple Gas Chromatograph-Mass Spectrometer (GC-MS TQ8030, Shimadzu, Japan). GC was performed with split ratio of 20:1 using helium as carrier gas (flow rate = 1 ml/min) with injection temperature of 300 °C. The column oven temperature was maintained at 60 °C with hold time of 5 min followed by increase to 280 °C with 8 °C/min ramp with a final hold of 37 min. The MS analysis was carried out in 70 eV electron ionization mode. To carry out MS, ion source temperature was 230 °C, interface temperature was 310 °C with a mass range of 45–600. The analysis of chromatograms was carried out by GC-MS solution software (version 4) and NIST 11 library database was used for compound identification^55^.

#### pH analysis

pH of the formation water was analyzed by digital pH meter (Gold 533, Digital Instruments Corporation, India).

#### Analysis of metals in formation water

The presence of the metals as Fe, Cu, Cr, Zn, Ni, Pb, Na, Mn, Ca, K and Mg in formation water was determined using atomic absorption spectroscopy (ASC-7000, Shimadzu, Japan) as per Ejike *et al*.^56^. For this, 100 mL of the formation water was subjected to nitric acid treatment with nitric acid before carrying out spectrometric analysis. Before the analysis, three calibrations using metal solution standards of a range of concentrations was carried out.

#### Analysis of chemical oxygen demand (COD) of formation water

The COD of the formation water sample was analyzed as per Trivedy and Goel^57^. For COD analysis, 20 mL of formation water sample was taken in a 250 mL COD flask followed by addition of 10 mL of 0.25 N potassium dichromate solution. To this, a pinch of Ag_2_SO_4_ and HgSO_4_ was added followed by addition of 30 mL sulphuric acid. The sample was refluxed for a period of 2 hours in COD reflux assembly. Following the reflux step, the sample was cooled and the final volume of the sample was made up to 140 mL with distilled water. To this 2–3 drops of ferroin indicator was added, mixed and titrated with 0.1 N ferrous ammonium sulphate. A distilled water sample was used as blank for the experiments. The COD was calculated as per the following equation:

$${\rm{COD}}({\rm{mg}}\,{{\rm{L}}}^{-1})=({\rm{b}}-{\rm{a}})\times {\rm{N}}\,{\rm{of}}\,{\rm{Ferrous}}\,{\rm{ammonium}}\,{\rm{sulphate}}\times 8000/{\rm{mL}}\,{\rm{sample}}$$

where,

a = volume of ferrous ammonium sulphate (mL) used for blank

b = volume of ferrous ammonium sulphate (mL) used for sample

8000 = miliequivalent weight of oxygen × 1000 mL/L

#### Analysis of SO_4_ in the formation water

The analysis of SO_4_ in the formation water was carried out by Turbidimetric method as per Trivedy and Goel^57^. For SO_4_ analysis, 5 mL of conditioning reagent (mixture of 75 g NaCl, 30 mL conc. HCl, 100 mL of 95% ethyl or isopropyl alcohol in 300 mL distilled water with addition of 50 mL glycerol) was added to 100 mL of the formation water sample. The sample was stirred on magnetic stirrer and a spoonful of BaCl_2_ crystals was added during stirring. After addition of the BaCl_2_ crystals, the sample is stirred for 1 minute and spectrophotometric reading of the sample at 420 nm was taken after 4 minutes. The concentration of SO_4_ in the formation water sample was determined from the standard curve. The standard curve (R^2^ = 0.99) was prepared by dissolving anhydrous Na_2_SO_4_ in distilled water in the concentration range of 0.0 to 100 mg L^−1^.

#### Analysis of nitrogen content in formation water

The total nitrogen was analyzed by taking 40 mL of the formation water sample in a 100 mL Kjeldahl flask to which 4 mL H_2_SO_4_, 10 drops of CuSO_4_ solution, 6 gm solid potassium sulphate and 1 mL of 10% NaCl solution was added. The flask was heated on a heater and as the water boils off, the sample turns dark due to decomposition of organic matter by H_2_SO_4_. As the digestion proceeds, the sample color turns pale green and the heating was continued for an additional 30 minutes. The flask was cooled and volume made up to 100 mL. 25 mL of this digest was distilled. A distilled water sample was used as a blank for the experiment. The distillate (in boric acid + mixed indicator) was titrated with 0.01 N HCl until the color changes from blue to brown or faint pink. The N content was calculated according to the following equation:

$${\rm{N}}\,({\rm{mg}}/{\rm{L}})=({\rm{a}}-{\rm{b}})\times {\rm{Normality}}\,{\rm{of}}\,{\rm{HCl}}\times 1000\times 14\times {\rm{D}}/{\rm{mL}}\,{\rm{sample}}\,{\rm{distilled}}.$$

where,

a = volume of HCl (mL) used with sample^57^

b = volume of HCl (mL) used with blank

1000 = conversion factor (mL/L)

14 = Atomic weight of Nitrogen

D = dilution factor (2.5). The original volume (40 mL) of sample has been made to 100 mL

after digestion.

### Isolation and molecular identification of algal strain from oil field formation water

The isolation of algal species present in oil field formation water was carried out as per standard isolation methodology described in Anderson^58^. The enrichment culture of algal species present in oil field formation water was carried out in Fog’s media supplemented with the antibiotic mix (Penicillin G, Dihydrosteptomycin sulfate and chloramphenicol) in standard dose (0.5 mL antibiotic mix to 50 mL algal medium) in order to avoid bacterial contamination in the cultures. To achieve this, 5 mL of oil field formation water was inoculated to the above-mentioned culture medium followed by incubation under illumination of 3500 lux (for photoperiod of 14 hours light: 10 hours dark) at orbital shaking of 110 rpm. The composition of Fog’s medium used in enrichment culture was as follows: MgSO_4_ (0.2 gm L^−1^), K_2_HPO_4_ (0.2 gm L^−1^), CaCl_2_ (0.1 gm L^−1^), Fe-EDTA solution (5 mL), KNO_3_ (0.2%) and 1 mL of micronutrient solution comprising of: H_3_BO_3_ (286 mg L^−1^), MnCl_2_. 4 H_2_O (181.0 mg L^−1^), ZnSO_4_. 7 H_2_O (22 mg L^−1^), Na_2_MoO_4_. 2H_2_O (39 mg L^−1^), CuSO_4_.5H_2_O (8 mg L^−1^)^59,60^. The pH of the medium was adjusted to 7.5. The enrichment step is followed by the dilution technique which is effective and is routinely used for isolating dominant algal species from a sample^58^. For this, 1 mL of the algal culture was used for serial dilution upto 10^−6^. For isolation of single algal colonies, 20 µL of the last diluted sample was streaked on solid Fog’s medium (containing the antibiotic mix) and incubated under illumination of 3500 lux for a photoperiod of 14 hours light: 10 hours dark. A loopful of the distinctly separated algal colony was inoculated to Fog’s medium and cultivated in an orbital shaking incubator under the same culture conditions as described above. The purity and morphological characteristics of the algal culture was studied by optical microscopic observation at 100X magnification.

The molecular identification of the algal culture was carried out by 18S rRNA based method. For this, the algal DNA was isolated and its quality evaluated on 1.0% agarose gel. The fragment of 18S rRNA region was amplified by PCR. The PCR amplicon was purified to remove contaminants. The forward and reverse DNA sequencing reaction of PCR amplicon was carried out with NS1 and NS4 primers using BDT v3.1 Cycle sequencing kit on ABI 3730xl Genetic Analyzer. The consensus sequence of the PCR amplicon was generated from forward and reverse sequence data using aligner software. The 18S rRNA region sequence was used to carry out BLAST with the database of NCBI GenBank. Based on maximum identity score first ten sequences were selected and aligned using multiple alignment software program Clustal W. Distance matrix was generated was constructed using MEGA 7.

### Algal growth kinetics and remediation of oil field formation water

In order to verify the capability of the algal isolate for remediation, its growth and hydrocarbon remediation efficiency in formation water was studied. To analyze the growth kinetics of the isolated alga in oil field formation water, different inoculums (500 mg L^−1^ 1000 mg L^−1^, 1500 mg L^−1^, 2000 mg L^−1^) of log phase algal culture was inoculated to respective Erlenmeyer flasks containing autoclaved oil field formation water. The inoculated flasks were incubated under the same culture conditions as described above. The growth of algal biomass in oil field formation water was monitored at regular intervals of 24 hours by optical density measurement at 560 nm in UV-Visible Spectrophotometer (UV-1800, Shimadzu, Japan) as well as by dry weight analysis^30,58^. The specific growth rate (µ) of the algal isolate was estimated by fitting a linear function to exponential phase of ln x(t) versus time curve, where x = biomass (mg/l) and t = time (days)^9^. For the control experiment, the algal inoculum of 1500 mg L^−1^ was inoculated to Erlenmeyer flask containing Fog’s medium and incubated under the same culture conditions as described above. The biomass growth as well as specific growth rate for the control cultures was analyzed as mentioned above. In order to verify the differences in chlorophyll A concentration between formation water cultivated and control biomass, the chorophyll extraction was performed as per Cuaresma *et al*.^61^. Two mL of the algal culture was subjected to centrifugation at 4400 rpm for time period of 6 minutes followed by addition of methanol to the resulting algal pellet after centrifugation. For algal pellet disruption, the samples were introduced to an ultrasound bath for 5 minutes followed by incubation for 40 minutes at 60 °C. Then, the sample was introduced to a temperature shock at 0 °C for 15 minutes. Then the samples are centrifuged and absorbance of the supernatant at 652 nm and 665 nm was analyzed in UV-Visible spectrophotometer (UV-1800, Shimadzu, Japan). The chlorophyll A concentration in the sample was determined using modified Arnon’s equations as:

$${{\rm{Chl}}}_{{\rm{a}}}=(\mathrm{34.9.}\,{{\rm{A}}}_{652}-\mathrm{15.28.}\,{{\rm{A}}}_{665}).{\rm{dilution}}\,{\rm{factor}}\,({{\rm{mgL}}}^{-1})$$

In order to elucidate the potential of the algal strain for remediation of hydrocarbons in formation water, the residual TPH in formation water was determined at a regular interval of 24 hours in the test experiments described above. To analyze this, the culture was centrifuged at 10000 rpm for a time period of 10 minutes and the cell-free supernatant obtained was analyzed for residual TPH content. The residual TPH was extracted by solvent extraction method and quantified by gravimetric analysis as described above. The hydrocarbon degradation percentage was estimated as described by Patowary *et al*.^55^:

$$\begin{array}{c}{\rm{Hydrocarbon}}\,{\rm{degradation}}( \% )=\\ \frac{({\rm{Weight}}\,{\rm{of}}\,{\rm{residual}}\,{\rm{TPH}}\,{\rm{in}}\,{\rm{abiotic}}\,{\rm{control}}-{\rm{Weight}}\,{\rm{of}}\,{\rm{residual}}\,{\rm{TPH}}\,{\rm{in}}\,{\rm{the}}\,{\rm{test}}\,{\rm{sample}})}{{\rm{Original}}\,{\rm{weight}}\,{\rm{of}}\,{\rm{TPH}}\,{\rm{introduced}}}\times 100\end{array}$$

The GC-MS analysis of the residual petroleum hydrocarbon fractions at the end of the experiment was analyzed as per the methodology described above for the test experiment which showed highest TPH removal from formation water. In order to analyze any loss of hydrocarbons due to abiotic factors, the oil field formation water without inoculation of the algae was incubated under the same culture conditions as mentioned above. For the abiotic control, the TPH was extracted by solvent extraction method followed by its gravimetric quantification and GC-MS analysis as described above. To verify if the algal growth is dependent on mixotrophism (utilize both hydrocarbon and inorganic carbon by photosynthesis as carbon source) or if the algal strain could utilize hydrocarbon as sole source of carbon, the algal strain was cultivalted in oil field formation water under dark condition. The growth of algal biomass, residual TPH and specific growth rate (µ) was determined as described above. To verify the role of sorption in TPH dissipation, biomass of *Chlorella vulgaris* BS1 was heat killed by autoclaving followed by incubation of dead biomass in formation water under the same experimental condition as described above for experiments with live cells. The residual TPH at equal intervals of 24 hours was analyzed as mentioned above. The inoculum concentration of dead biomass for the experiment was taken as 3500 mg L^−1^ (since 3500 mg L^−1^ biomass is obtained after incubation of 1 day with starting algal inoculum of 1500 mg L^−1^ as mentioned in Fig. 2c). This allows comparison between the amount of TPH dissipated after 1 day of incubation between dead biomass (sorption) and live cells (biodegradation) which will help verify if TPH dissipation is mainly contributed by sorption or biodegradation.

Concomitantly with TPH remediation, any improvement in other physico-chemical parameters determining formation water quality as pH, COD, ionic content and heavy metals was analyzed for the test experiment which showed highest TPH removal from formation water. For this, the culture was centrifuged (10000 rpm for 10 minutes) to obtain the cell-free supernatant followed by its analysis as per standard methodology described above.

To analyze if the algal isolate *Chlorella vulgaris* BS1 adapts to saline stress by a mechanism involving enhanced carotenoid biosynthesis, the differences in carotenoid content between formation water cultivated and control biomass was estimated. For this, an equal volume of biomass from both the culture conditions was harvested by centrifugation at 4400 rpm for 6 minutes. This was followed by addition of methanol to the algal biomass. For cell disruption, the samples were subjected to an ultrasound bath treatment of 5 minutes and then incubated at temperature of 60 ^o^C for 40 minutes. After this, the samples were exposed to a temperature shock at 0 °C for 15 minutes. For analysis, the samples were centrifuged followed by estimation of the absorbance of the resulting supernatant at 470 nm in UV-Visible spectrophotometer (UV-1800, Shimadzu, Japan). Then modified Arnon’s equation was used to determine the carotenoid content of the sample^61^. The carotenoid content in algal biomass was expressed as per gram of biomass (dry weight).

In relation to the adaptive response of the algal strain to grow under saline stress in formation water, formation water quality parameters determining phycoremediation efficiency as salinity, Total Dissolved Solids (TDS), Total Suspended Solids (TSS), Total Organic Carbon (TOC) and Sodium Adsorption Ratio (SAR) was determined. Salinity and Total Dissolved solids (TDS) was analyzed using multiparameter water quality meter (HI 98194, Hanna Instruments, USA). Total Organic Carbon (TOC) was analyzed using TOC analyzer (Shimadzu, Japan). The gravimetric determination of Total Suspended Solids (TSS) as per Method 2540 D of APHA^62^. A glass- fiber filter is placed on the filter support screen of the filtration apparatus and rinsed with three successive volumes of ≥30 mL distilled water. The vacuum was kept on until all traces of water is removed from the filter. The filter is dryed at 103–105 °C for a time period of 60 minutes and then its weight is recorded. A known volume of well-mixed sample was then filtered by the pre-weighed filter with vacuum applied. The surface area of the exposed filter was successively rinsed for three times with ≥10 mL distilled water. The vacuum is kept on until the filter is free from all traces of water. The filter is dried at 103–105 °C for 60 minutes and record its weight. The drying cycle was repeated until a constant weight was achieved. The concentration of TSS was calculated as per the following equation:

$${\rm{TSS}}\,({\rm{mg}}\,{{\rm{L}}}^{-1})=({\rm{A}}-{\rm{F}})\times 1000/{\rm{S}}$$

Where

A = final weight of dried residue + filter (mg)

F = pre-weigh of filter (mg)

S = sample volume (mL)

Sodium Adsorption Ratio (SAR) is calculated as^52^:

$${\rm{SAR}}=\frac{{\rm{Na}}}{\sqrt{\frac{({\rm{Ca}}+{\rm{Mg}})}{2}}}$$

All the ionic concentrations are in meq L^−1^.

### Statistical analysis

All experiments were carried out in triplicates and the results were represented as mean ± standard deviation. One-way analysis of variance (ANOVA) with Turkey HSD test was performed to analyze significant differences of TPH degradation in formation water following treatment with various inoculum of algae as well in abiotic control. Origin Pro8 (Origin Lab Corporation, Northampton, MA, USA) was used to carry out the statistical analysis.

## Supplementary information

Supplementary Information
