# Supplementary material for: A cost-effective and environmentally sustainable process for phycoremediation of oil field formation water for its safe disposal and reuse
Source: Sci Rep. 2019 Oct 23;9:15232. doi: 10.1038/s41598-019-51806-5 (PMC6811566; doi:10.1038/s41598-019-51806-5)
Supplement: Supplementary file 1 — Supplementary Information [file 41598_2019_51806_MOESM1_ESM.pdf]

A cost-effective and environmentally sustainable process for phycoremediation of oil field formation water for its safe disposal and reuse

Bhaskar Das , Suresh Deka

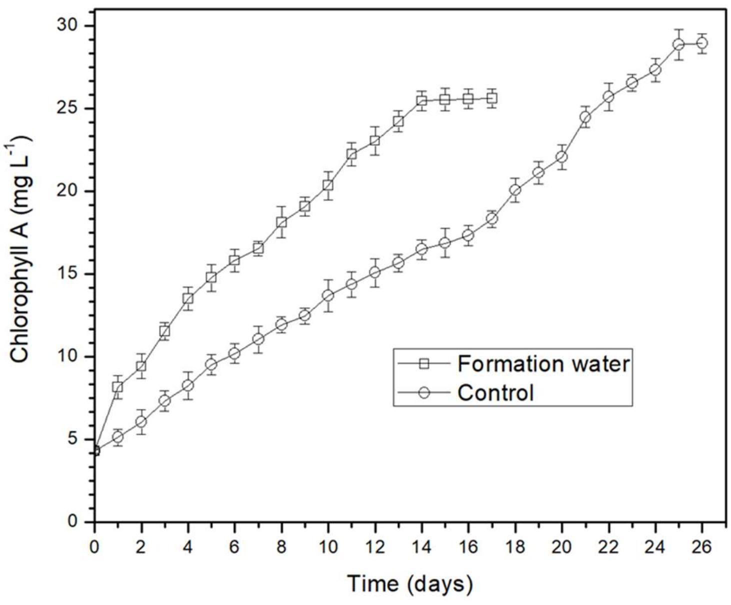

Supplementary Figure 1. Chlorophyll profile of *Chlorella vulgaris* BS1 cultivated in oil field formation water and control cultures.

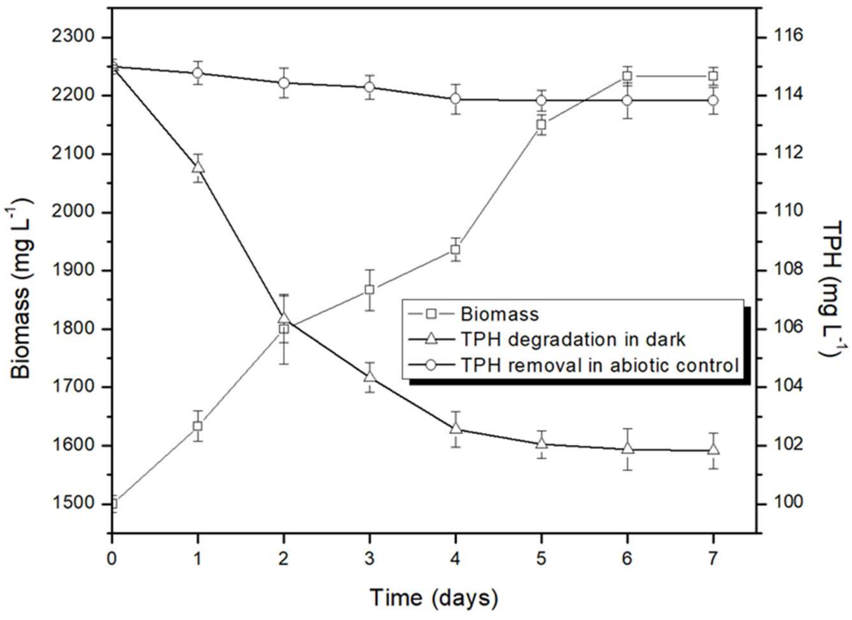

Supplementary Figure 2 Biomass growth and TPH degradation profile of *Chlorella vulgaris* BS1 in dark

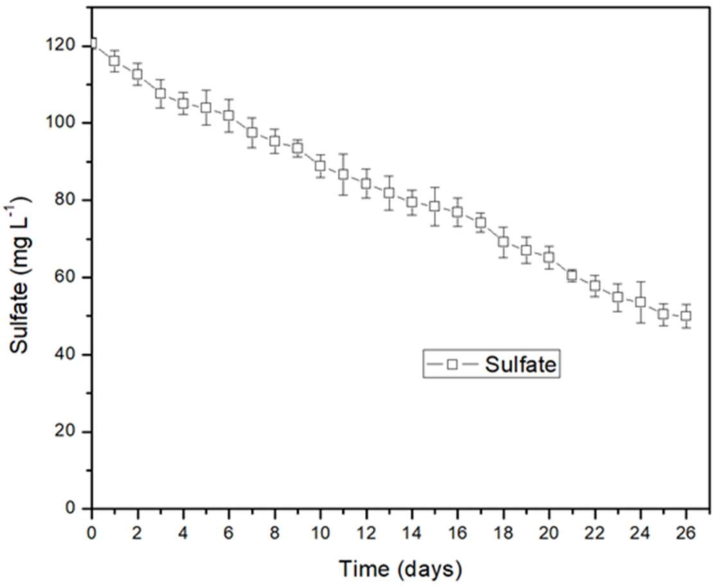

Supplementary Figure 3. Sulfate uptake profile of *Chlorella vulgaris* BS1 in control culture.

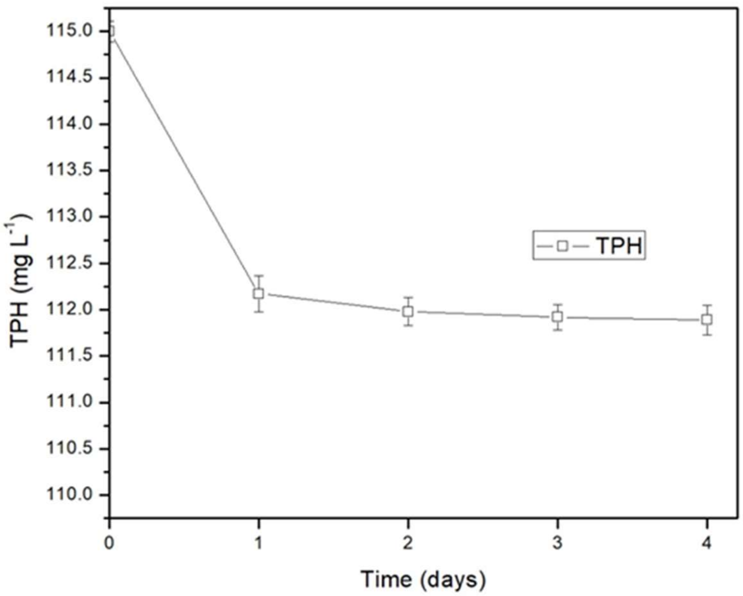

Supplementary Figure 4 TPH sorption by dead biomass of *Chlorella vulgaris* BS1

Supplementary Table 1. Estimates of evolutionary divergence between sequences

**Sample_P3**		0.001	0.001	0.001	0.001	0.002	0.002	0.002	0.002	0.002	0.002
FR865683.1	0.002		0.000	0.000	0.000	0.001	0.001	0.001	0.001	0.001	0.001
HQ111432.1	0.002	0.000		0.000	0.000	0.001	0.001	0.001	0.001	0.001	0.001
GQ487225.1	0.002	0.000	0.000		0.000	0.001	0.001	0.001	0.001	0.001	0.001
GQ487223.1	0.002	0.000	0.000	0.000		0.001	0.001	0.001	0.001	0.001	0.001
KY364701.1	0.003	0.001	0.001	0.001	0.001		0.001	0.001	0.001	0.001	0.001
KP726221.1	0.003	0.001	0.001	0.001	0.001	0.002		0.000	0.001	0.001	0.001
LK021940.1	0.003	0.001	0.001	0.001	0.001	0.002	0.000		0.001	0.001	0.001
HQ111435.1	0.003	0.001	0.001	0.001	0.001	0.002	0.002	0.002		0.001	0.001
HQ111434.1	0.003	0.001	0.001	0.001	0.001	0.002	0.002	0.002	0.002		0.001
GQ867590.1	0.003	0.001	0.001	0.001	0.001	0.002	0.002	0.002	0.002	0.002	

Supplementary Table 2. Residual TPH concentration (mg L^{-1}) in formation water after treatment with *Chlorella vulgaris* BS1

	**Incubation time (days)**		
**Inoculum (mg/l)**	**0**	**7**	**14**
500	115 ± 0.12^{a}	90.04 ± 0.23 ^{b}	54.91 ± 0.32 ^{c}
1000	115 ± 0.15^{a}	75.9 ± 0.32 ^{d}	25.13 ± 0.27 ^{e}
1500	115 ± 0.11^{a}	59.93 ± 0.16 ^{f}	0.04 ± 0.12 ^{g}
2000	115 ± 0.17^{a}	66.48 ± 0.34 ^{h}	5.81 ± 0.13 ^{i}
Abiotic Control	115 ± 0.18^{a}	113.68 ± 0.14 ^{j}	113.46 ± 0.29 ^{j}

TPH degradation values are average of three replicates and described in terms of residual TPH concentration (mg L^{-1}). Different alphabets (a to j) as prefix of Mean ± SD values in the same row and column denote statistically significant differences as per Turkey HSD p-value ($p<0.01$) while same alphabets in the same row or column indicates no significant difference as per Turkey HSD p-value (Turkey HSD inference $p<0.01$)

Supplementary Table 3. Comparison of TPH profile in oil field formation water treated with *Chlorella vulgaris* BS1 and untreated abiotic control

Compounds	Retention Time (min)	Molecular formula	Molar Mass (g mol^{-1})	Abiotic control formation water after 14 days incubation (%)	Formation water treated for 14 days by *Chlorella vulgaris* BS1 (%)
Dodecane	13.305	$C_{12}H_{26}$	170.34	0.09	Not Detected
Decane, 1,1'-oxybis-	14.802	$C_{20}H_{42}O$	298.5469	0.07	Not Detected
11-Methyldodecanol	15.249	$C_{13}H_{28}O$	207.409	1.14	Not Detected
Tetradecane	16.664	$C_{14}H_{30}$	198.394	0.85	Not Detected
1-Dodecanol	17.761	$C_{12}H_{26}O$	186.339	6.15	Not Detected
Eicosane	18.048	$C_{20}H_{42}$	282.556	0.18	Not Detected
Pentadecane	18.165	$C_{15}H_{32}$	212.421	0.65	Not Detected
Phenol, 2,4-bis(1,1-dimethylethyl	18.288	$C_{14}H_{22}O$	206.329	7.42	Not Detected
1-Decanol, 2-hexyl-	18.592	$C_{16}H_{34}O$	242.447	0.92	Not Detected
2,4-Dimethyldodecane	18.906	$C_{14}H_{30}$	198.394	0.11	Not Detected
Heptadecane	19.070	$C_{17}H_{36}$	240.475	2.93	Not Detected
Pentadecane, 8-hexyl-	19.175	$C_{21}H_{44}$	296.583	0.35	Not Detected
Heptadecane, 8-methyl-	20.185	$C_{18}H_{38}$	254.502	0.13	Not Detected
Hentriacontane	20.223	$C_{31}H_{64}$	436.853	0.51	Not Detected

Dodecane, 4-methyl-	20.357	$C_{13}H_{28}$	184.367	0.34	Not Detected
Heneicosane	20.432	$C_{21}H_{44}$	296.583	0.48	Not Detected
Heptadecane, 2-methyl-	20.537	$C_{18}H_{38}$	254.502	0.43	Not Detected
Tetrapentacontane	20.699	$C_{54}H_{110}$	759.474	0.70	Not Detected
Hexadecane	20.975	$C_{16}H_{34}$	226.448	3.02	Not Detected
2-methylhexacosane	21.213	$C_{27}H_{56}$	380.745	0.42	Not Detected
Tetrapentacontane, 1,54-dibromo	21.272	$C_{54}H_{108}Br_2$	917.266	0.48	Not Detected
Nonahexacontanoic acid	21.574	$C_{69}H_{138}O_2$	999.861	1.07	Not Detected
Heneicosane	22.189	$C_{21}H_{44}$	296.583	3.39	Not Detected
2-methylhexacosane	22.444	$C_{27}H_{56}$	380.745	0.56	Not Detected
Hexadecane, 1-iodo-	22.959	$C_{16}H_{33}I$	352.344	0.19	Not Detected
Cyclohexane, (1-octylnonyl)-	23.054	$C_{23}H_{46}$	322.6113	0.45	Not Detected
5,5,7,7-Tetraethylundecane	23.224	$C_{19}H_{40}$	268.529	0.12	Not Detected
Nonane, 5-(2-methylpropyl)-	23.534	$C_{13}H_{28}$	184.367	0.12	Not Detected
Hexatriacontyl pentafluoropropionate	24.935	$C_{39}H_{73}F_5O_2$	668.9877	0.26	Not Detected
Heptadecane, 4-methyl-	25.203	$C_{18}H_{38}$	254.502	0.20	Not Detected
Dodecylcyclohexane	25.441	$C_{18}H_{36}$	252.486	0.10	Not Detected
Sulfurous acid, cyclohexylmethyl	25.892	$C_{13}H_{26}O_3S$	276.44	0.07	Not Detected

2-methylhexacosane	25.983	$C_{27}H_{56}$	380.745	0.56	Not Detected
Octadecanoic acid	26.313	$C_{18}H_{36}O_2$	284.484	2.29	Not Detected
Hexacontane	26.438	$C_{60}H_{122}$	843.636	0.43	Not Detected
1-Hentetracontanol	26.555	$C_{41}H_{84}O$	593.122	0.44	Not Detected
Octatriacontyl trifluoroacetate	26.824	$C_{40}H_{77}F_3O_2$	647.049	0.14	Not Detected
1-Naphthalenamine, N-phenyl-	27.234	$C_{16}H_{13}N$	219.287	2.81	Not Detected
Cyclohexane, decyl-	27.618	$C_{16}H_{32}$	224.432	0.06	Not Detected
Nonadecane, 2,6,10,14-tetram	27.889	$C_{22}H_{46}$	310.61	0.20	Not Detected
4-Methyldocosane	27.985	$C_{23}H_{48}$	324.637	0.17	Not Detected
Hexanedioic acid, bis(2-ethylhexyl	28.600	$C_{22}H_{42}O_4$	370.574	0.10	Not Detected
Dotriacontane	28.727	$C_{32}H_{66}$	450.88	1.6	Not Detected
2-Nonadecanone, O-methyloxime	29.270	$C_{20}H_{41}NO$	311.554	0.08	Not Detected
Octadecane, 3-ethyl-5-(2-ethylbutyl	29.731	$C_{26}H_{54}$	366.718	0.11	Not Detected
Bis(2-ethylhexyl) phthalate	29.972	$C_{24}H_{38}O_4$	390.564	0.64	Not Detected
5,5-Diethylheptadecane	30.088	$C_{21}H_{44}$	296.5741	0.33	Not Detected
5,5-Diethylpentadecane	31.900	$C_{19}H_{40}$	268.52	0.11	Not Detected
Tetracosane	32.297	$C_{24}H_{50}$	338.6538	0.25	Not Detected
Hexadecanoic acid, tetradecyl	33.774	$C_{30}H_{60}O_2$	452.808	0.80	Not Detected
